# Borderline personality disorder and violence in the UK population: categorical and dimensional trait assessment

**DOI:** 10.1186/s12888-016-0885-7

**Published:** 2016-06-03

**Authors:** Rafael A. González, Artemis Igoumenou, Constantinos Kallis, Jeremy W. Coid

**Affiliations:** Centre for Mental Health, Division of Brain Sciences, Department of Medicine, Imperial College, London, UK; Center for Evaluation and Sociomedical Research, Graduate School of Public Health, University of Puerto Rico, Puerto Rico, USA; Violence Prevention Research Unit, Queen Mary University of London, London, UK

**Keywords:** Borderline personality disorder, Violence, Aggression, Intimate partner violence, Dimensional, Personality disorders

## Abstract

**Background:**

Borderline personality disorder (BPD) is characterised by difficulties with impulse control and affective dysregulation. It is unclear whether BPD contributes to the perpetration of violence or whether this is explained by comorbidity. We explored independent associations between categorical and dimensional representations of BPD and violence in the general population, and differential associations from individual BPD criteria.

**Methods:**

We used a representative combined sample of 14,753 men and women from two British national surveys of adults (≥16 years). BPD was assessed using the Structured Clinical Interview II- Questionnaire. We measured self-reported violent behaviour in the past 5 years, including severity, victims and locations of incidents. Associations for binary, dimensional and trait-level exposures were performed using weighted logistic regression, adjusted for demography and comorbid psychopathology.

**Results:**

Categorical diagnosis of BPD was associated only with intimate partner violence (IPV). Associations with serious violence leading to injuries and repetitive violence were better explained by comorbid substance misuse, anxiety and antisocial personality disorder (ASPD). However, *anger* and *impulsivity* BPD items were independently associated with most violent outcomes including severity, repetition and injury; *suicidal behaviours* and *affective instability* were not associated with violence. Both trait-level and severity-dimensional analyses showed that BPD symptoms might impact males and females differently in terms of violence.

**Conclusions:**

For individuals diagnosed BPD, violence is better explained by comorbidity. However, BPD individual traits show different pathways to violence at the population level. Gender differences in BPD traits and their severity indicate distinct, underlying mechanisms towards violence. BPD and traits should be evaluated in perpetrators of IPV.

## Background

Borderline personality disorder (BPD) is characterised by difficulties with impulse control, affective dysregulation and unstable patterns of relating to others particularly in close relationships [1]. These personality traits can be observed in other psychiatric disorders associated with violence in the general population, such as substance misuse, antisocial personality (disorder) and anxiety disorders [2]. Personality disorder (PD) cluster B symptoms are prevalent among men who perpetrate partner violence, and violent persons have been found to exhibit persistence of PD traits over time [3]. Nevertheless, it is unclear whether BPD has a direct role in the perpetration of violence that is not due to coexisting psychopathology [4].

BPD is overrepresented in clinical and forensic criminal justice settings [5, 6], many of whom are convicted for serious violent crimes [7] and engage in higher rates of aggression when comorbid with ASPD [8]. Males and females convicted for domestic violence offenses report significantly higher borderline traits [9, 10], which is consistent with the patterns of volatile relationships that characterize their personality pathology. However, using prospective data from psychiatric patients in the MacArthur Study of violence [11], BPD did not predict serious violence or aggressive behaviour *after* adjustments for comorbid ASPD and psychopathy [12].

One shortcoming when making conclusions based on prison and clinical samples is that they represent individuals at the extreme end of a spectrum of severity and may differ from community samples on important variables other than their PD. These studies may be unrepresentative of rates of violence associated with BPD in the general population. Currently, population and community studies on independent contributions of BPD to violence are scarce, have included small samples, with inconclusive results. Nevertheless, research [13] reported that despite univariable models showing associations with aggression, these were better explained by substance misuse and paranoid PD traits. Meanwhile, based on another community sample, BPD was associated with self- reported psychological and physical partner aggression above and beyond ASPD [14]. Moderation by gender was reported in association with aggression favouring women. However, generalization of these findings remains limited because the study was on older adults (55 to 64 years).

Because of the heterogeneity of BPD symptoms, a binary classification could include symptoms that are both conducive to and deter violence. Considering dimensional models of assessing personality disorders has been strongly debated for inclusion in the last two editions of the DSM classification system, with several prominent PD researchers arguing in favour of, and putting forth alternative systems [15–18]. Broadening the scope of assessment to include dimensional and trait-level (i.e., symptoms) approaches for measuring correlates of PD may be preferable for several reasons: it allows quantification of severity based on trait and areas of dysfunction, it increases power to test moderation by subgroups such as gender, and when considering individual trait analyses, it allows us to partial out the specific correlates of each components of BPD with violence. For example, when using a dimensional approach with community subjects, researchers reported that while BPD and ASPD both share a common link to impulsivity, they are differentially related to emotional and physical aggression [19]. This approach allows the identification of specific traits, such as dysregulation or self-injurious behaviours that may be associated with violence after accounting for demographic and clinical confounders, together with whether any of the individual BPD items maybe protective of violence.

In the present study, we overcome previous limitations by reporting on violence in a representative household sample from Great Britain of over 14,000 men and women. Our primary aims were to estimate the association of categorical and dimensional representations of BPD with violence in the population, including level of severity, victim types and location of events. Confounding from psychiatric morbidity was considered on all models to estimate the independent role of BPD in violence. To estimate differential impact on violence from specific BPD diagnostic criteria, we also examined items independently. The moderating role of gender on associations with violence was additionally investigated.

## Method

### Design and sample

The sample was drawn from the first phase of the ONS Survey of Psychiatric Morbidity among Adults in Great Britain (2000) and the Adult Psychiatry Morbidity Survey (2007), two British national surveys of adults aged 16 years and older living in households in England, Scotland and Wales in 2000, and in England in 2007. A total of 8580 adults completed a first-phase interview (response rate 69.5 %) in the 2000 survey and 7393 in 2007 (response rate 57.0 %). Design and sampling procedures have been previously described [20, 21]. Of the total sample, 1220 (7.6 %) were excluded due to missing data on the exposure (BPD) and on the violent behaviours module. Because each survey employed the same measures of PD, demography, clinical covariates and violence outcomes, we conducted joint analyses of individual-level data for a total weighted sample of 14,753.

### Measures

#### Measurement of PD

Borderline and Antisocial personality disorders were identified using the Structured Clinical Interview for DSM-IV *patient questionnaire* (SCID-II screen) [22]. Participants gave “yes” or “no” responses to questions in order to screen for the categories of DSM-IV Axis-II included in this study (i.e., ASPD and BPD) [1]. Cut-off points were manipulated to increase levels of agreement, measured by the Kappa coefficient, between both individual criteria and clinical diagnoses as previously reported [23]. These same algorithms were used in the present survey for categorical classifications.

Nine discrete DSM-IV based BPD criteria were derived from the SCID-II questionnaire: (1) Frantic efforts to avoid real or imagined abandonment (*avoid abandonment*), (2) Unstable and intense interpersonal relationships (*unstable relationships*), (3) Identity disturbance: markedly and persistently unstable (*identity disturbance*), (4) Impulsivity in at least 2 areas (*impulsivity*), (5) Recurrent suicidal behaviour or gestures (*suicidal behavior*), (6) Affective instability due to a marked reactivity of mood (*affective instability*), (7) Chronic feelings of emptiness (*emptiness*), (8) Inappropriate, intense anger (*anger*), and (9) Transient, stress-related paranoid ideation (*paranoid ideation*).

The dimensional BPD traits measure is a severity scale based on the number of BPD criteria endorsed. This dimensional transformation of BPD criteria was originally presented by Widiger and colleagues [16, 18] arguing in favour of an alternative system for the DSM-IV. The system was subsequently proposed by Oldham & Skodol [15] and more recently discussed in Skodol et al. [24]; PD is described according to six points on a scale based on the number of criteria met: absent traits = 0; clinically significant traits = 1, 2, or 3; sub-threshold traits = 4; threshold of the disorder = 5; pervasive disorder = 6, 7, or 8; and prototypic disorder = 9.

ASPD positive screening was used as a covariate in adjusted models. No other PD categories were used in the study.

#### Measurement of clinical syndromes

Participants were also screened for presence of psychosis when any 3 of 5 criteria from the Psychosis Screening Questionnaire (PSQ) [25] were present. Furthermore, using the revised version of the Clinical Interview Schedule (CIS-R) [26] diagnoses of common mental disorders were combined into a single category of anxiety disorder from five subcategories, including mixed anxiety/depressive disorder, generalised anxiety disorder (GAD), panic disorder, phobic disorder, and obsessive compulsive disorder (OCD).

Alcohol dependence was identified on the basis of responses to two questionnaires, the Alcohol Use Disorders Identification Test (AUDIT) [27] and the community version of the Severity of Alcohol Dependence Questionnaire (SADQ-C) [28]. All respondents with an AUDIT score of 10 or more were also interviewed with the SADQ-C. A score of four or more on the latter was taken to indicate dependence.

Participants who in the past year had used cannabis, amphetamines, crack, cocaine, ecstasy, tranquillisers, opiates or volatile substances were asked five questions designed to assess drug dependence based on the Diagnostic Interview Schedule [29]. Endorsement of any of the questions in relation to any of these substances in the past year was used to indicate drug dependence [21].

#### Measurement of violent behaviour

All participants were queried regarding their violent behaviour in the context of establishing a diagnosis of ASPD. However, as we retained ASPD as a covariate in adjusted models, we used additional questions to measure violent behaviour. Similar to previous literature reporting on surveys from New York and Israel, [30, 31] participants were asked the initial question: “Have you been in a physical fight, assaulted, or deliberately hit anyone in the past 5 years?” If respondents replied positively, additional questions covered location of incidents (the perpetrator’s home, someone else’s home, the street or other outdoor location, a bar, the workplace, some other place), victim type (a spouse or partner, a family member, a friend, some other known person, a stranger, a police officer, some other person not covered), and outcome of the incident. Spouses or cohabiting partners and girlfriends or boyfriends were combined into a single category of “intimate partner violence”. We defined self-reported violent behaviour as severe if victim or respondent were injured; and as repetitive if respondents had been involved in five or more violent incidents over the previous 5 years. We also asked whether there had been injuries as a result of the violence act, and whether it occurred under the influence of alcohol and/or drugs.

#### Social class classification

Social class was based on the UK Registrar General’s Classification [32] which uses the most recent occupation of the head of household: I – professional, II –managerial, IIIA – skilled manual, IIIB – skilled non-manual, IV – partly skilled, V – unskilled. These were combined in three categories: I & II (upper middle class), III (lower middle and skilled working class) and IV & V (less skilled and unskilled). This classification aptly represents income, education, and level of responsibility at work [33].

#### Analytic strategy

For descriptive purposes, absolute (n) and relative frequencies (%) were reported for all dichotomous and multi-categorical variables. Contrasts for proportions of each BPD criterion by gender were estimated using F-distribution tests for complex survey samples.

Logistic regression models were used to estimate associations between BPD and each violent outcome. Odds Ratios (OR) with 95 % confidence intervals (CI) were used to represent the magnitude of the associations. Models for the association of BPD and violence are presented unadjusted and adjusted for age, gender, marital status, social class, ethnicity, and clinical covariates including: drug and alcohol dependence, psychosis, anxiety disorder and ASPD.

The independent associations between each BPD criterion with the violent outcomes were examined by adjusting the logistic regression models for all other BPD criteria simultaneously. Independent BPD criteria models were performed for the total sample and stratified by gender.

For the dimensional traits model, we regressed ‘Any violence in the past 5 years’ on the ordinal BPD dimensional measure- a test of linear trend- adjusted for demographic characteristics and clinical covariates. We tested for effect- modification of gender on the linear association between the dimensional BPD measure and violence.

Data were weighted to adjust for the effects of selecting one individual per household and under-representation of certain subgroups, and to account for any deviation from selecting a simple random sample. All models employed robust standard errors to adjust for clustering of individuals within postcodes. A binary variable based on the source of data (i.e., 2000 or 2007 survey) was included as a fixed covariate on all models.

An α level of <0.05 was adopted throughout the study. All analyses were performed using Stata version 13 (StataCorp.).

## Results

Multivariate models were fitted to examine associations between selected demographic and clinical covariates with the main violence outcome, and with BPD separately. Being male (OR 3.46 CI 95 % [2.92, 4.11], *p* <0.001), single (OR 1.72 CI 95 % [1.43, 2.06], *p* <0.001) or separated (OR 2.00 CI 95 % [1.58, 2.52], *p* <0.001), and from social classes below I&II (OR 2.05 CI 95 % [1.69, 2.47], *p* <0.001) were all associated with increased likelihood of violence. Being older than 34 (OR 0.33 CI 95 % [0.28, 0.40], *p* <0.001), and from Indian subcontinent ethnicity (OR 0.48 CI 95 % [0.26, 0.87], *p* <0.05) were less likely to report violence. All psychiatric covariates including drug (OR 2.36 CI 95 % [1.76, 3.15], *p* <0.001) and alcohol dependence (OR 2.20 CI 95 % [1.75, 2.76], *p* <0.001), anxiety disorder (OR 1.96 CI 95 % [1.63, 2.36], *p* <0.001), and ASPD (OR 2.75 CI 95 % [2.01, 3.76], *p* <0.001) increased the odds of violence in adjusted models, except for positive psychosis screening, which had a protective association (OR 0.28 CI 95 % [0.10, 0.74],*p* <0.05).

The prevalence of BPD did not differ by gender. Being younger, separated or divorced, and from lower social classes were all associated with BPD. All categories of psychiatric morbidity were associated with BPD, with particularly large effects sizes (Table 1).

**Table 1:** Clinical syndromes in association with BPD classification

	Borderline Personality Disorder, *n* = 219 (1.5 %)			
Covariates	n	%	OR (95 % CI)	AOR (95 % CI)^a^
Drug dependence	53	9.8	9.28 (6.49, 13.27)***	2.74 (1.66, 4.52)***
Alcohol dependence	73	7.2	7.40 (5.21, 10.51)***	2.75 (1.78, 4.26)***
Anxiety disorder	163	7.3	17.83 (12.58, 25.26)***	11.75 (7.98, 17.32)***
Psychosis	12	28.7	27.54 (12.99, 58.41)***	6.44 (2.68, 15.51)***
ASPD	57	11.3	11.62 (8.05, 16.76)***	3.01 (1.80, 5.05)***

Note. Weighted percentages (row) and estimates (*N* = 14,753)

^a^Adjusted for gender, age, marital status, social class and ethnicity, and all psychiatric morbidity

*p < 0.05,**p <0.01,***p <0.001

Table 2 shows univariate and multivariate models of BPD with all characteristics, victim types and locations of violence. After adjustments for demographic covariates and psychiatric morbidity, associations with BPD were restricted to intimate partners and violence taking place in their home.

**Table 2:** Univariate and multivariate models of association between BPD classification and violence outcomes in a joint UK household survey (*N* = 14,753)

	BPD SCID-II				
Outcomes	n (%)	OR	95 % CI	AOR^a^	95 % CI
Any violence	80 (36.5)	5.39	3.87,7.49***	1.47	0.92,2.36
Intoxicated	52 (23.9)	7.38	5.01,10.88***	1.61	0.90,2.89
Minor violence	17 (7.6)	1.96	1.07,3.58*	0.91	0.46,1.82
Severity					
5 > incidents	25 (11.3)	6.48	3.92,10.70***	1.63	0.82,3.25
Victim injured	26 (11.7)	4.13	2.59,6.60***	0.71	0.37,1.34
Victim types					
IPV	32 (14.7)	11.79	7.53,18.46***	1.90	1.03,3.51*
Family member^b^	8 (3.7)	3.87	1.84,8.17***	1.28	0.51,3.21
Friend	22 (10.1)	5.96	3.45,10.29***	1.30	0.57,2.95
Person known	27 (12.3)	4.39	2.68,7.19***	0.97	0.49,1.92
Stranger	34 (15.7)	3.76	2.38,5.92***	1.01	0.54,1.87
Locations					
Own home	32 (14.7)	9.98	6.35,15.67***	2.17	1.19,3.94*
Street	53 (24.0)	5.52	3.84,7.93***	1.33	0.77,2.32
Bar/pub	37 (16.8)	6.13	3.94,9.52***	1.47	0.78,2.77

Note. Weighted percentages (column) and estimates

^a^Adjusted for gender, age, marital status, social class and ethnicity; drug and alcohol dependence, psychosis, ASPD and anxiety disorder

^b^Not adjusted for psychosis due to complete separation (i.e., non-exposed cases perfectly predict outcome)

*p < 0.05, **p <0.01, ***p <0.001

### Individual BPD criteria and violence

Women endorsed a significantly higher proportion of several BPD criteria than men: *avoid abandonment*, *unstable relationships*, *identity disturbance*, *affective instability* and *emptiness* (Table 3).

**Table 3:** Frequency distributions of each BPD and dimensional criteria by gender and total sample

	Females		Males			
BPD DSM-IV Criteria	n	%	n	%	F^a^	*p*
1 Frantic efforts to avoid real or imagined abandonment	1707	22.7	1356	18.7	26.9	<0.001
2 Unstable and intense interpersonal relationships	1582	21.1	1379	19.1	7.0	0.008
3 Identity disturbance: markedly and persistently unstable	157	2.1	112	1.6	4.3	0.04
4 Impulsivity in at least 2 areas	2871	38.2	2875	39.7	2.8	0.09
5 Recurrent suicidal behaviour, gestures	218	2.9	173	2.4	2.5	0.11
6 Affective instability due to a marked reactivity of mood	1291	17.2	859	11.9	61.8	<0.001
7 Chronic feelings of emptiness	1378	18.3	999	13.8	44.9	<0.001
8 Inappropriate, intense anger	587	7.8	580	8.0	0.2	0.69
9 Transient, stress-related paranoid ideation	482	6.4	465	6.4	0.0	0.98
Dimensional traits of DSM-IV BPD					3.8	0.002
1 Traits absent	3062	40.7	3102	42.9		
2 Clinically significant traits	3589	47.8	3459	47.8		
3 Sub-threshold traits	344	4.6	276	3.8		
4 Threshold of the disorder	250	3.3	167	2.3		
5 Pervasive disorder	259	3.4	216	3.0		
6 Prototypic disorder	12	0.2	17	0.2		

Note. Weighted percentages (column) and estimates (*N* = 14,753)

^a^F distribution test of association for survey data (Stata Corp. *svyset* commands)

Table 4 shows that criterion 2 (*unstable relationships*) was only independently associated with IPV. Criteria 5, 6 and 9 (*suicidal behaviour*, *affective instability and paranoid ideation*, respectively) were not directly associated with any of the violent outcomes. Criterion 3 (*identity disturbance*) was associated with fewer reports of minor violence and violence towards persons known to them.

**Table 4:** Adjusted^a^ (independent) associations between each BPD criterion and violence outcomes in a joint UK household survey (*N* = 14,753)

	Borderline personality disorder DSM-IV criteria								
	Avoid abandonment	Unstable relationships	Identity disturbance	Impulsivity	Suicidal behaviour	Affective instability	Emptiness	Anger	Paranoid ideation
Outcomes	AOR(95 % CI)	AOR(95 % CI)	AOR(95 % CI)	AOR(95%CI)	AOR(95%CI)	AOR(95 % CI)	AOR(95 % CI)	AOR(95 % CI)	AOR(95 % CI)
Any violence	1.33 (1.10–1.60)**	1.15 (0.94–1.40)	0.69 (0.40–1.17)	1.46 (1.24–1.71)***	1.09 (0.74–1.61)	1.17 (0.90–1.51)	0.87 (0.68–1.12)	2.47 (1.92–3.19)***	1.18 (0.90–1.55)
Intoxicated	1.12 (0.84–1.50)	1.15 (0.85–1.55)	1.03 (0.55–1.96)	1.20 (0.94–1.53)	0.91 (0.55–1.50)	1.07 (0.74–1.55)	0.95 (0.65–1.39)	2.07 (1.44–2.97)***	1.27 (0.88–1.83)
Minor violence	1.28 (0.99–1.65)	1.25 (0.95–1.66)	0.30 (0.13–0.67)**	1.63 (1.29–2.07)***	0.80 (0.47–1.37)	1.11 (0.78–1.58)	1.00 (0.70–1.43)	1.77 (1.22–2.58)**	1.41 (0.95–2.07)
Severity									
5 > incidents	0.95 (0.66–1.38)	1.29 (0.87–1.91)	0.94 (0.47–1.89)	1.72 (1.18–2.50)**	1.09 (0.58–2.05)	1.06 (0.64–1.75)	0.70 (0.42–1.15)	3.38 (2.14–5.34)***	1.03 (0.60–1.76)
Victim injured	1.16 (0.84–1.60)	0.80 (0.56–1.14)	0.89 (0.43–1.84)	1.44 (1.08–1.92)*	1.20 (0.68–2.12)	1.04 (0.68–1.59)	0.99 (0.66–1.48)	2.41 (1.63–3.58)***	0.82 (0.51–1.32)
Victim types									
IPV	2.17 (1.56–3.00)***	1.47 (1.01–2.14)*	0.76 (0.39–1.47)	1.40 (1.00–1.97)*	1.18 (0.68–2.05)	1.13 (0.75–1.71)	0.79 (0.51–1.22)	3.36 (2.22–5.09)***	1.15 (0.73–1.81)
Family^b^	1.01 (0.58–1.75)	0.96 (0.52–1.77)	0.96 (0.37–2.47)	2.00 (1.22–3.28)**	1.63 (0.70–3.80)	1.35 (0.60–3.06)	1.28 (0.63–2.57)	1.57 (0.75–3.28)	0.93 (0.43–2.02)
Friend	1.37 (0.90–2.09)	1.47 (0.95–2.29)	0.48 (0.21–1.11)	1.31 (0.89–1.94)	1.41 (0.75–2.65)	0.87 (0.50–1.53)	0.72 (0.41–1.26)	2.57 (1.53–4.29)***	1.37 (0.78–2.39)
Person known	1.69 (1.24–2.31)***	1.23 (0.89–1.70)	0.34 (0.17–0.68)**	1.75 (1.31–2.34)***	0.66 (0.36–1.21)	0.94 (0.61–1.44)	1.22 (0.81–1.83)	1.82 (1.21–2.74)**	1.21 (0.80–1.82)
Stranger	1.14 (0.88–1.48)	0.87 (0.65–1.16)	1.20 (0.61–2.37)	1.41 (1.12–1.77)**	1.15 (0.70–1.89)	1.04 (0.71–1.52)	0.69 (0.47–1.02)	1.98 (1.43–2.74)***	0.99 (0.66–1.49)
Locations									
Own home	1.35 (0.99–1.85)	1.24 (0.87–1.79)	1.05 (0.57–1.95)	1.59 (1.14–2.20)**	1.22 (0.70–2.13)	1.31 (0.84–2.06)	1.32 (0.88–2.00)	2.62 (1.72–3.99)***	0.80 (0.51–1.25)
Street	1.36 (1.06–1.74)*	1.07 (0.82–1.40)	0.77 (0.42–1.43)	1.54 (1.23–1.93)***	0.96 (0.61–1.50)	0.86 (0.62–1.20)	0.79 (0.56–1.10)	2.44 (1.81–3.30)***	1.14 (0.81–1.62)
Bar/pub	1.41 (1.05–1.89)*	0.77 (0.56–1.07)	0.81 (0.44–1.50)	1.44 (1.09–1.91)*	0.65 (0.39–1.10)	1.22 (0.82–1.84)	0.88 (0.58–1.33)	2.02 (1.38–2.95)***	1.27 (0.81–1.98)

Note. Weighted Logistic Regression estimates

^a^Adjusted for gender, age, marital status, social class and ethnicity; drug and alcohol dependence, psychosis, ASPD and anxiety disorder

^b^Not adjusted for psychosis due to complete separation (i.e., non-exposed cases perfectly predict outcome)

*p < 0.05, **p < 0.01, ***p < 0.001

In contrast, *anger* was significantly associated with all violent outcomes except towards family members. *Impulsivity* was associated with violence repetition, victim injury and minor incidents, towards intimate partners, family members and other known persons, in their own homes, as well as outdoors and in bars/pubs. There were associations between *avoid abandonment* and violence towards intimate partners, known persons, and in all locations except the participants’ home.

### Individual BPD criteria by gender

Stratified analyses of BPD criteria by gender revealed further differences. For males, *paranoid ideation* was associated with all key violent outcomes: violence when intoxicated (OR 2.07 CI 95 % [1.38, 3.10], *p* <0.001), repetitive violence (OR 2.91 CI 95 % [1.69, 5.01], *p* <0.001), violence leading to injuries (OR 2.19 CI 95 % [1.37, 3.50], *p* <0.001) and minor incidents of violence(OR 1.70 CI 95 % [1.05, 2.76], *p*<0.05). However, among women it was not associated with any of these outcomes. In women, *unstable relationships* was associated with violence (OR 1.44 CI 95 % [1.04, 2.01], *p* <0.05), but mostly of minor consequence (OR 1.81 CI 95 % [1.16, 2.83], *p* <0.01) and towards intimate partners (OR 1.59 CI 95 % [1.03, 2.45], *p* <0.05). There were no associations among males for this criterion.

For both men and women, the *impulsivity* criterion was similarly associated with most violent outcomes.

### Dimensional BPD traits and violence: differences by gender

The adjusted linear association of the dimensional (severity) trait scale was significant (OR 1.42 CI 95 % [1.31, 1.54], *p* <0.001). A gender X BPD dimensional traits interaction term in this model was significant (OR 0.86 CI 95 % [0.75, 0.98],*p* <0.05), indicating that the linear increase between BPD traits and violence is significantly higher in women (OR 1.56 CI 95 % [1.41, 1.72], *p* <0.001) compared with men (OR 1.34 CI 95 % [1.20, 1.49], *p* <0.001).

## Discussion

Our results were based on a uniquely large and representative sample of the UK population and showed that a categorical representation of BPD was only associated with intimate partner violence (IPV). Associations found with serious violence leading to injuries and repetition were better explained by comorbid psychopathology. Specific BPD traits varied in their magnitude and direction of associations, with the *anger* and *impulsivity* criterions associated with most outcomes including violence severity, repetition and injury. However, *suicidal behaviours* and *affective instability* were not associated with violence. Both trait-level and dimensional-severity analyses revealed that BPD symptoms may affect males and females differently in terms of violence.

For those with a categorical diagnosis of BPD, violence was accounted for by coexisting ASPD, anxiety disorders and substance use disorders. The association between BPD and violence towards intimate partners was expected, and is consistent with previous research findings [3, 14, 34]. BPD is characterised by unstable relationships, and this corresponds to the demographic finding that violence was more common among those separated from their partners. Under stress, individuals with BPD can also experience transient paranoid ideation [1]. It is possible that with intimate partners, persons with BPD may manifest persecutory ideas and episodes of extreme jealousy.

### Individual BPD symptoms and violence

BPD is a complex disorder which is frequently correlated with other risk factors for violence [35]. In the present study, it was highly comorbid with all other categories of mental and emotional distress, including anxiety and psychotic symptoms. Analyses were therefore performed at the individual criteria level. These revealed differential correlates between violence and individual BPD criteria. As expected, *Anger* and *impulsivity* were associated with higher risk for most violent outcomes. *Anger* was more than twice as likely to be associated with repetitive violence and with violence directed at intimate partners. A caveat is required as one of the questions that assesses the BPD SCID-II *anger* criterion is “Do you hit people or throw things when you get angry?”, indirectly measuring aggressiveness.

*Avoid abandonment* was also directly linked to the outcomes, with violence limited towards intimate partners or persons known, and in which the perpetrator was injured. This would suggest violent altercations had resulted from perceived rejection and acting under threat of being abandoned. Taken together with the disproportionately high rates of comorbidity with anxiety, it is likely that these individuals may have experienced insecure-anxious attachments in early life which had continued into adulthood in their close relationships [36]. Insecure-anxious attachments refer to the perceived availability of attachment figures, thought to develop through inconsistent patterns of early care. Under high levels of stress, they frequently amplify the severity of their adversities, become obsessed with thoughts of being abandoned by partners, with accompanying intense negative emotions [37, 38].

Our analyses showed that several BPD traits are independently linked with violence. BPD is a very heterogeneous disorder and includes symptoms across cognitive, affective, interpersonal and impulse control domains [39]. That different BPD traits were associated with violence, such as *anger*, *impulsivity*, and *avoid abandonment* is consistent with the assertion that violence related to PD is driven conceptually by a combination of high expressions of internalising and externalising traits, especially in the context of co-occurring ASPD [35]. Comorbidity between ASPD and BPD is high [40], and this perspective especially accounts for the wide heterogeneity of violence perpetration, taking place in various contexts (for in depth review, see Howard, 2015 [35]). Moreover, the different correlates of violence from BPD traits appear to operate differentially by the externalising and internalising aspects of BPD pathology, and in turn, the internalising pathway appears more prominently represented in women.

*Suicidal behavior*, *affective instability* and *emptiness* were not independently associated with any violent outcomes. These traits represent the depressive-mood dimension of BPD. Previous research has shown that depressive disorders are associated with reduction of violence both in clinical samples [41, 42] and at the population level [43]. In the present study, these BPD traits were not protective. However, they may have cancelled out or attenuated the effects of other criteria when using the categorical classification for analysis. Although *suicidal behaviour* is an aggressive act towards the self and may occur in the context of impulsivity, it was not associated with interpersonal violence in this study. Self-directed violence is consistent with a model of depression in which aggression is turned inwards [44]. Our findings may therefore indicate contrasting emotional and behavioural dynamics for outwardly expressed violence. Finally, the *identity disturbance* criterion was inversely associated with violence, indicating a protective effect.

### Differences by gender

Our data shows that BPD traits were differentially associated with violence for men and women. Stratification shows that women who endorsed *unstable relationships* were significantly more prone to engage in minor violence, directed at their partners. However, there were no associations with violence among men and *unstable relationships*. Certain traits were associated with violence for both men and women, including *anger*, *impulsivity*, and to a lesser extent *avoid abandonment*. However, male violence was strongly associated with *paranoid ideation* when under stress. This suggests a potential mechanism for interpersonal violence perpetuated by men, characterised by general suspiciousness and jealousy within intimate relationships. The link between this borderline personality trait and violence resembles the ‘BD’ subtype described by Holtzworth-Munroe et al. [34] in their typology of male batterers where violent men are thought to have deficits in their marriage and relationships skills, embrace hostile attitudes towards women, and are characterised by extreme jealousy. It is hypothesised that these men may have experienced early trauma and abuse, with a destructive effect on their personality organization, which leads to intimate partner violence. For example, men endorsing the *paranoid ideation* trait were almost four times as likely to be violent towards intimate partners in our study, lending support to this typology. This is also consistent with recent findings indicating that the pathway from early abuse to IPV is mediated by personality disorder amongst young men [45]. However, an alternative explanation is that this personality trait overlaps with the suspiciousness criterion of other cluster B personality disorders.

We found that change in the linear increase of BPD symptoms on violence is higher among women in contrast to men. This indicates that women are more susceptible to becoming violent with increasing BPD traits, as this increase reduces the base rate gender differences in violence in our sample. This symptom by gender interaction shows that women are more prone to engage in violence due to abnormal personality traits, specifically BPD. Conversely, for men, BPD traits showed a more linear (stable) association, with a higher base rate for violence.

### Limitations

The population sample used in this study is to our understanding the largest used for evaluating the association of BPD and violence, which ensures generalizability. This allowed us to test complex models while minimizing estimate bias. However, the present findings should to be considered in the context of several limitations. Firstly, diagnostic categories in this survey were derived from the structured SCID-II screening questionnaire, which was administered by lay interviewers without clinical training. It is likely that the SCID-II screen resulted in a number of false positives, a problem with self-report questionnaires when making PD diagnoses [46]. Despite this limitation, our reported BPD prevalence of 1.5 % is remarkably similar to the 1.4 % reported in the National Comorbidity Survey Replication [47], and slightly lower than the revised NESARC prevalence [48].

Another important limitation was that the associations between PD and violence may include content overlap (shared variance). Violent behaviour forms part of the criteria for conduct disorder (initiating physical fights, using a weapon) and also among items occurring after the age of 15 (irritability), which may coexist with BPD criteria. The use of self-reported violence was another limitation. Although self-report is commonly used to investigate general and partner violence, evidence suggests that subjects tend to under-report aggression [49], although reliance on official statistics including criminal records would result in substantially greater underreporting of all forms of violence except the most serious. Finally, the cross-sectional nature of the design limits any conclusions regarding causality between BPD traits and BPD as a categorical construct in its association with violence.

Nevertheless, the present study further demonstrates the value of assessing underlying traits directly, thereby lending support to emerging models that focus on basic personality features discussed in the literature [17, 24].

### Implications

Our finding that a clinical diagnosis of BPD was not independently associated with violence outside of intimate partner relationships has important implications for treatment and management. BPD is associated with stigma and patients have been excluded from services in the past. Correspondingly, BPD has not been found to be independently associated with violent or sexual convictions among prisoners [50]. Nevertheless, BPD is highly prevalent among correctional populations and we found that it is associated with violence when comorbid with other psychiatric conditions in the general population. This indicates firstly, the need for further research into the mechanisms of how BPD interacts with comorbid psychopathology, together with the possibility that it has synergistic effects with certain conditions. Secondly, that BPD mediates between certain risk factors and violence. For instance, BPD traits have been shown to mediate between childhood maltreatment and IPV [51]. Thirdly, BPD and certain risk factors for violence may have a common aetiology.

Our findings of a strong and independent relationship between BPD and IPV must be placed in the context of our previous findings from this combined UK community sample. Self-reported violence towards partners was more prevalent among women than men. However, the risks of becoming physically injured in a violent altercation with a partner were considerably higher among women than men [52]. A small but important community study has emphasised the importance of using dyadic couples in studying the associations between BPD traits and IPV, and has confirmed previous research showing that each partners’ personality traits can influence the other’s [53]. More BPD traits among men were associated with more violence towards partners and victimisation by their partners, whereas women’s BPD traits were associated only with their victimisation [54]. Our findings are therefore important in demonstrating that BPD traits among women in this larger community sample were also associated with violence directed at their partners, but that there are also differential associations between BPD traits and violence between men and women. These differences must also be placed in the context of additional findings from our sample: very few men were exclusively violent towards their partners [52]. Violence within relationships is commonly bidirectional and these findings demonstrate why violence directed against women by men carries higher risks and is more persistent, together with why strategies to treat men have been less successful. BPD traits among violent men are therefore important in contexts outside of their relationships and can include violence towards strangers. A narrow approach towards violence within relationships is therefore unlikely to be effective when treating men.

Our findings have additional importance both for treatment of women with BPD and women who are violent towards partners. More information is needed on the prevalence of violence in clinical samples of BPD and this may be a relatively unrecognised problem compared to self-harming behaviour. Clinicians should therefore ask about violence towards others among both male and female patients with BPD. However, assertions that women’s violence towards partners is more likely to be motivated by self-defence and fear whereas men’s is more likely to be associated with control motives [55] may be simplistic and unhelpful in the assessment of IPV, particularly in the treatment of women with BPD and violent women with BPD traits. More complex factors of fear of abandonment and associated states of severe anxiety, which in turn are associated with early disordered attachments that have persisted into adulthood, indicate specific goals for treatment, together with the need for support for partners to maintain supportive relationships.

## Conclusions

The independent association between BPD with intimate partner violence (IPV) can be interpreted in the context of problematic anxious attachment, which may offer routes for addressing in psychotherapeutic treatment programmes. Our study is the first to partial out the direct contribution of the different components of BPD towards violence, revealing distinct pathways from high levels of internalizing and externalizing psychopathology inherent to this complex disorder. Gender differences in BPD traits and their severity indicate distinct, underlying mechanisms towards violence that should be examined in future studies.

## Abbreviations

APMS 2007, adult psychiatry morbidity survey; ASPD, antisocial personality disorder; AUDIT, alcohol use disorders identification test; BPD, borderline personality disorder; CIS-R, clinical interview schedule; DSM-IV, diagnostic and statistical manual of mental disorders, 4^th^ edition; GAD, generalised anxiety disorder; IPV, intimate partner violence; LRECs, Local Research Ethics Committees; MREC, Multi-Centre Research Ethics Committee; OCD, obsessive compulsive disorder; ONS, Office for National Statistics; PD, personality disorder; PSQ, psychosis screening questionnaire; SADQ-C, severity of alcohol dependence questionnaire
